# Chronic physical conditions, physical multimorbidity, and quality of life among adults aged ≥ 50 years from six low- and middle-income countries

**DOI:** 10.1007/s11136-022-03317-6

**Published:** 2022-12-26

**Authors:** Olawale Olanrewaju, Mike Trott, Lee Smith, Guillermo F. López Sánchez, Christina Carmichael, Hans Oh, Felipe Schuch, Louis Jacob, Nicola Veronese, Pinar Soysal, Jae Il Shin, Laurie Butler, Yvonne Barnett, Ai Koyanagi

**Affiliations:** 1 Cambridge Centre for Health, Performance, and Wellbeing, Anglia Ruskin University, Cambridge, CB1 1PT UK; 2 Centre for Public Health, Queens University Belfast, Belfast, UK; 3 Division of Preventive Medicine and Public Health, Department of Public Health Sciences, School of Medicine, University of Murcia, Murcia, Spain; 4 Suzanne Dworak Peck School of Social Work, University of Southern California, Los Angeles, CA 90007 USA; 5 Department of Sports Methods and Techniques, Federal University of Santa Maria, Santa Maria, Brazil; 6 Research and Development Unit, Parc Sanitari Sant Joan de Déu, CIBERSAM, ISCIII, Dr. Antoni Pujadas, 42, Sant Boi de Llobregat, 08830 Barcelona, Spain; 7 Department of Internal Medicine and Geriatrics, University of Palermo, 90133 Palermo, Italy; 8 Department of Geriatric Medicine, Bezmialem Vakif University, 34093 Istanbul, Turkey; 9 Department of Pediatrics, Yonsei University College of Medicine, Yonsei-Ro 50, Seodaemun-Gu, C.P.O Box 8044, Seoul, 03722 Republic of Korea; 10 ICREA, Pg, Lluis Companys 23, 08010 Barcelona, Spain; 11 Faculty of Health Sciences, Universidad Autónoma de Chile, Providencia, Chile

**Keywords:** Multimorbidity, Quality of life, Older adults, Low- and middle-income countries

## Abstract

**Purpose:**

Multimorbidity (i.e., ≥ 2 chronic conditions) poses a challenge for health systems and governments, globally. Several studies have found inverse associations between multimorbidity and quality of life (QoL). However, there is a paucity of studies from low- and middle-income countries (LMICs), especially among the older population, as well as studies examining mediating factors in this association. Thus, the present study aimed to explore the associations, and mediating factors, between multimorbidity and QoL among older adults in LMICs.

**Methods:**

Cross-sectional nationally representative data from the Study on Global Ageing and Adult Health were analyzed. A total of 11 chronic conditions were assessed. QoL was assessed with the 8-item WHO QoL instrument (range 0–100) with higher scores representing better QoL. Multivariable linear regression and mediation analyses were conducted to assess associations.

**Results:**

The final sample consisted of 34,129 adults aged ≥ 50 years [mean (SD) age 62.4 (16.0) years; age range 50–114 years; 52.0% females]. Compared to no chronic conditions, 2 (b-coefficient − 5.89; 95% CI − 6.83, − 4.95), 3 (− 8.35; − 9.63, − 7.06), 4 (− 10.87; − 12.37, − 9.36), and ≥ 5 (− 13.48; − 15.91, − 11.06) chronic conditions were significantly associated with lower QoL, dose-dependently. The mediation analysis showed that mobility (47.9%) explained the largest proportion of the association between multimorbidity and QoL, followed by pain/discomfort (43.5%), sleep/energy (35.0%), negative affect (31.9%), cognition (20.2%), self-care (17.0%), and interpersonal activities (12.0%).

**Conclusion:**

A greater number of chronic conditions was associated with lower QoL dose-dependently among older adults in LMICs. Public health and medical practitioners should aim to address the identified mediators to improve QoL in patients with multimorbidity.

**Supplementary Information:**

The online version contains supplementary material available at 10.1007/s11136-022-03317-6.

## Introduction

Multimorbidity can be defined as the presence of two or more chronic conditions [1, 2]. By 2035, it has been reported that about 17% of the population in the United Kingdom will have four or more chronic conditions [3]. The prevalence of multimorbidity is even higher in low- and middle-income countries (LMICs) [4, 5]. For example, data from the World Health Organization’s Study on Global Ageing and Adult Health (SAGE) found that the overall prevalence of multimorbidity among adults aged ≥ 18 years from six LMICs was 21.9% [6]. Although present across the life-course, the risk of multimorbidity increases with age, owing to older age per se being one of the most important risk factors for non-communicable diseases [2]. Also, people are living longer, albeit spending most of their later-life with ill-health and disability.

Multimorbidity is associated with considerable burden to the individual, population health and health systems [3]. Some of these burdens include increase in demand for health and social care, polypharmacy, the need for complex health and care management, poor health outcomes, increased functional dependence, and lower quality of life (QoL) [2, 7, 8]. In particular, QoL is a widely used measure in the evaluation of health-care services, patient-reported and population health outcomes. QoL is defined as the degree to which an individual is happy/comfortable, healthy, and able to participate in life events, and is often measured across multiple life domains (e.g., psychological, physical, social, environmental) [9]. There is evidence in the literature of inverse associations between multimorbidity and QoL [5, 10, 11]; however, the evidence is limited due to study heterogeneity and studies predominantly being based in high-income countries, while evidence on this subject is limited in LMIC settings, especially among the older population. A meta-analysis (*n* = 2,500,722) aimed at exploring the relationship between multimorbidity and QoL found that QoL, measured by the WHOQoL-BREF, decreased per disease added (− 4.37%; 95% CI − 7.13, − 1.61) [12]. However, only 18/74 studies included in this meta-analysis were from LMICs, and just 39% of the included studies were conducted in free-living/community dwelling-populations. Moreover, to the best of our knowledge, there are no multi-country studies on multimorbidity and QoL specifically on the older population. This indicates that more studies examining the multimorbidity/QoL relationship from the general older population of LMICs are needed since findings from high-income countries are unlikely to be generalizable to LMICs. For example, disease profiles may differ in LMICs, while multimorbidity can have a particularly negative impact on QoL in such settings due to limited availability of health care. In addition to this, to the best of our knowledge, there are no previous studies which have attempted to quantify the extent to which potential mediators such as mobility limitations, pain, sleep problems, or negative affect mediate the association between multimorbidity and QoL. This is an important research gap as this can potentially inform interventions that improve QoL among people with multimorbidity. Although Arokiasamy and colleagues (2015) conducted a similar study using the SAGE (which confirmed an inverse relationship with QoL), their study did not explore mediating factors [6], and examined all adults aged ≥ 18 years rather than focusing on the older population, despite multimorbidity being much more highly prevalent in the older population.

Therefore, the present study aimed to explore the association between multimorbidity and QoL in community-dwelling older populations in LMICs (China, Mexico, South Africa, Russia, India, and Ghana) using data from the SAGE. In addition, we tested whether and to what extent perceived health statuses such as pain, cognition, and mobility might mediate this association. We hypothesized that multimorbidity will be associated with lower levels of QoL, and that this association will be partly mediated by pain, mobility, and cognition.

## Methods

Data from the Study on Global Ageing and Adult Health (SAGE) were analyzed. These data are publicly available through https://www.who.int/data/data-collection-tools/study-on-global-ageing-and-adult-health.This survey was undertaken in China, Ghana, India, Mexico, Russia, and South Africa between 2007 and 2010. Based on the World Bank classification at the time of the survey, all countries were LMICs. Details of the survey methodology have been published elsewhere [13]. Briefly, in order to obtain nationally representative samples, a multistage clustered sampling design method was used. The sample consisted of adults aged ≥ 18 years with oversampling of those aged ≥ 50 years. Trained interviewers conducted face-to-face interviews using a standard questionnaire. Standard translation procedures were undertaken to ensure comparability between countries. The survey response rates were: China 93%; Ghana 81%; India 68%; Mexico 53%; Russia 83%; and South Africa 75%. Sampling weights were constructed to adjust for the population structure as reported by the United Nations Statistical Division. Ethical approval was obtained from the WHO Ethical Review Committee and local ethics research review boards. Written informed consent was obtained from all participants.

### Chronic physical conditions and physical multimorbidity

We included all 11 chronic physical conditions for which data were available in the SAGE. *Chronic back pain* was defined as having had back pain every day during the last 30 days. Respondents who answered affirmatively to the question “Have you lost all of your natural teeth?” were considered to have *edentulism*. The participant was considered to have *hearing problems* if the interviewer observed this condition during the survey. *Hypertension* was defined as having at least one of the following: systolic blood pressure ≥ 140 mmHg; diastolic blood pressure ≥ 90 mmHg; or self-reported diagnosis. *Visual difficulty* was defined as having severe/extreme difficulty in seeing and recognizing a person that the participant knows across the road [14]. *Diabetes* and *stroke* were solely based on lifetime self-reported diagnosis. For other conditions, the participant was considered to have the condition in the presence of either one of the following: self-reported diagnosis; or symptom-based diagnosis based on algorithms. We used these algorithms, which have been used in previous studies using the same dataset, to detect undiagnosed cases [15, 16]. Specifically, the validated Rose questionnaire was used for *angina* [17], and other previously validated symptom-based algorithms were used for *arthritis*, *asthma*, and *chronic lung disease* [15]. Further details on the definition of chronic physical conditions can be found in Table S1 (Appendix). The total number of chronic physical conditions was calculated and categorized as 0, 1, 2, 3, 4, and ≥ 5. Multimorbidity was defined as ≥ 2 chronic physical conditions, in line with previously used definitions [16].

### Quality of life (QoL)

The 8-item WHO Quality of Life (WHOQoL) instrument, which is a shortened version of the WHOQoL-BREF, was used to assess QoL. There were two questions each for four domains (i.e., physical, psychological, social, environmental) [18]. Participants answered each question rated on a five-point Likert scale ranging from 1 (not at all) to 5 (completely) or 1 (very dissatisfied) to 5 (very satisfied). A composite score was created by summing the responses of the different questions and rescaling the result from 0 to 100 with higher scores representing better QoL. Good internal consistency of this scale and acceptable convergent validity with WHOQoL-BREF have been reported [18, 19].

### Mediators

Seven factors related to health status that can be the consequence of multimorbidity, and also be the cause of lower QoL were selected as potential mediators [20–29]. Specifically, these health statuses in the past 30 days were evaluated with 14 health-related questions (i.e., two questions per domain) pertaining to seven domains including (i) mobility; (ii) self-care; (iii) pain/discomfort; (iv) cognition; (v) interpersonal activities; (vi) negative affect; (vii) sleep/energy. These domains have been used as indicators of health status in prior studies utilizing the same questions [30–32]. The actual questions can be found in Table S2 (Appendix). Each item was scored on a five-point scale ranging from ‘none’ to ‘extreme/cannot do’. For each separate domain, we used factor analysis with polychoric correlations to obtain a factor score which was later converted to scores ranging from 0 to 100 [30, 32] with higher values representing worse health function.

### Control variables

The selection of control variables was based on previous literature [12, 33] and included age, sex, highest level of education achieved (≤ primary, secondary, tertiary), wealth quintiles based on income, marital status (currently married/cohabiting, never married, separated/divorced/widowed), employment status (engaged in paid work ≥ 2 days in last 7 days: yes or no), social participation, physical activity, and smoking (never, current, past).

As in a previous SAGE publication [33], a social participation index was created based on nine questions on the participant’s involvement in community activities in the past 12 months with five answer options ranging from “never” to “daily”. The actual questions can be found in Table S3 (Appendix). The answers to these questions were summed and later converted to a scale ranging from 0 to 100 with higher scores corresponding to higher levels of social participation. Levels of physical activity were assessed with the Global Physical Activity Questionnaire and were classified as low, moderate, and high based on conventional cut-offs [34].

### Statistical analysis

The statistical analysis was done with Stata 14.2 (Stata Corp LP, College station, Texas). The analysis was restricted to those aged ≥ 50 years. The difference in sample characteristics between those with and without multimorbidity (i.e., ≥ 2 chronic conditions) was tested by Chi-squared tests and Student’s *t*-tests for categorical and continuous variables, respectively.

Multivariable linear regression analyses were used to assess the association between number of chronic conditions and individual chronic conditions (exposures) and QoL (outcome) using the overall sample. Country-wise analysis was also conducted, and this used multimorbidity as the exposure variable. To assess the degree of between-country heterogeneity in the association between multimorbidity and QoL, we calculated the Higgin’s *I*^2^ based on country-wise estimates. This represents the degree of heterogeneity that is not explained by sampling error with values of 25%, 50%, and 75% often being considered as low, moderate, and high levels of heterogeneity [35]. Overall estimates were obtained based on country-wise estimates by meta-analysis with random effects.

Next, in order to gain an understanding of the extent to which various factors related to health status may explain the relation between multimorbidity and QoL, we conducted mediation analysis using the *khb* (Karlson Holm Breen) command in Stata [36]. This method decomposes the total effect of a variable into direct and indirect effects (i.e., the mediational effect). Using this method, the percentage of the main association explained by the mediator can also be calculated (mediated percentage). Each potential mediator was included in the model individually.

The analysis on the number of chronic conditions and QoL was also stratified by age and sex. All regression analyses including the mediation analysis were adjusted for age, sex, education, wealth, marital status, unemployment, social participation, physical activity, smoking, and country, except for the country-wise and sex-stratified analyses, which were not adjusted for country and sex, respectively. The analysis with individual chronic conditions as the exposure variable mutually adjusted for all chronic conditions. Adjustment for country was done by including dummy variables for each country in the model as in previous SAGE publications. The sample weighting and the complex study design were considered in all analyses. Results from the regression analyses are presented as b-coefficients with 95% confidence intervals (CIs). The level of statistical significance was set at *P* < 0.05.

## Results

**Fig. 1:**
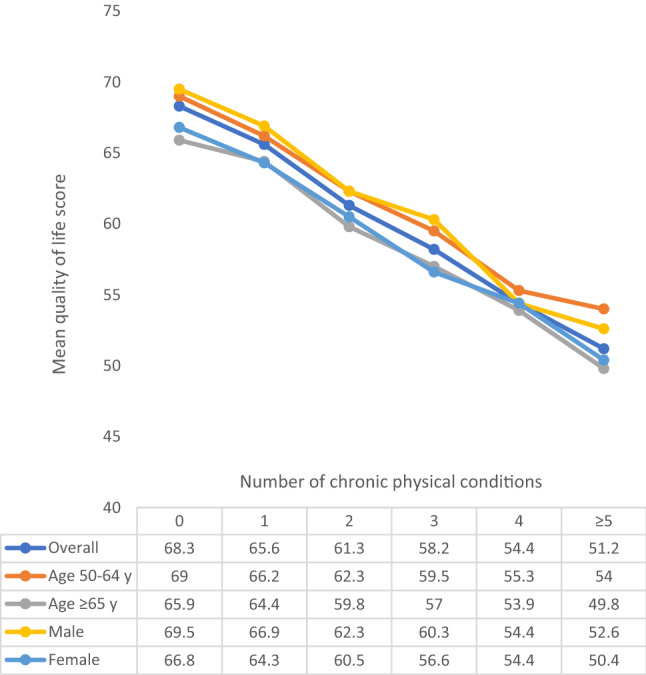
Mean quality of life score by number of chronic physical conditions. Quality of life was based on a scale ranging from 0 to 100 with higher scores representing better quality of life

**Fig. 2:**
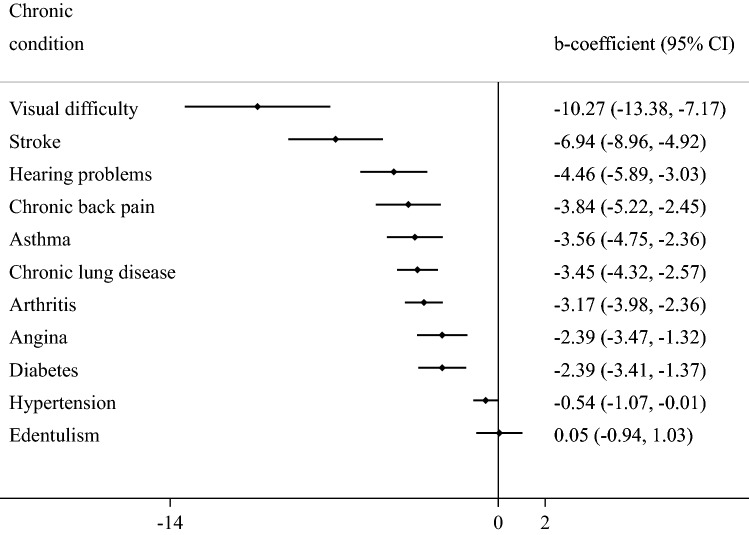
Association between individual chronic physical conditions and quality of life (outcome) estimated by multivariable linear regression. *CI* Confidence interval. Quality of life was based on a scale ranging from 0 to 100 with higher scores representing better quality of life. Models are mutually adjusted for all 11 individual chronic conditions, and age, sex, education, wealth, marital status, unemployment, social participation, physical activity, smoking, and country

**Fig. 3:**
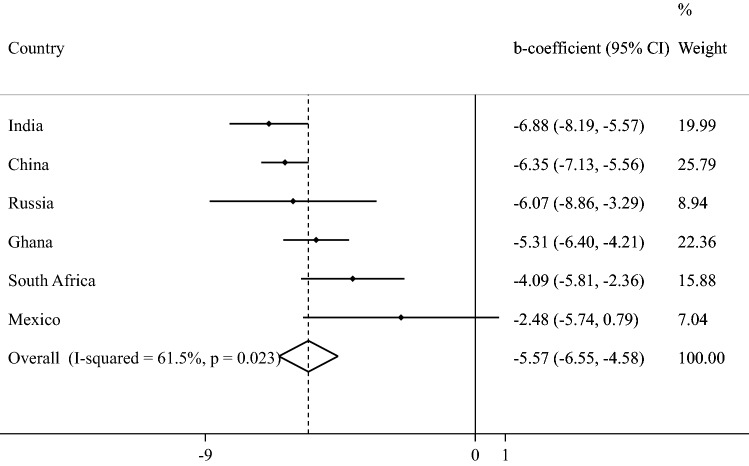
Country-wise association between physical multimorbidity (i.e., ≥ 2 chronic conditions) and quality of life estimated by multivariable linear regression. *CI* Confidence interval. Quality of life was based on a scale ranging from 0 to 100 with higher scores representing better quality of life. Models are adjusted for age, sex, education, wealth, marital status, unemployment, social participation, physical activity, and smoking. Overall estimate was obtained by meta-analysis with random effects

The final sample consisted of 34,129 adults aged ≥ 50 years [mean (SD) age 62.4 (16.0) years; age range 50–114 years; 52.0% females]. The sample size in each country were: China *n* = 13,175; Ghana *n* = 4305; India *n* = 6560; Mexico *n* = 2313; Russia *n* = 3938; South Africa *n* = 3838. The prevalence of 1, 2, 3, 4, and ≥ 5 chronic conditions was 32.3%, 22.4%, 12.2%, 6.4%, and 4.6%, respectively. The sample characteristics are provided in Table 1. The mean QoL score decreased sharply with increasing number of chronic conditions (Fig. 1). In terms of individual chronic conditions, all conditions assessed in the study were associated with significantly lower QoL, except for edentulism (Fig. 2). Adjusted analysis showed that compared to no chronic conditions, having greater number of chronic conditions is associated with significantly lower QoL scores dose-dependently with the b-coefficient (95% CI) of ≥ 5 conditions being − 13.48 (− 15.91, − 11.06) (Table 2). The estimates by age groups and sex were similar. Country-wise analysis showed that multimorbidity (i.e., ≥ 2 chronic conditions) is associated with lower QoL in all the six countries, although the estimate for Mexico was not statistically significant (Fig. 3). A moderate level of between-country heterogeneity was observed (*I*^2^ = 61.5%) with the overall estimate based on a meta-analysis being − 5.57 (95% CI = − 6.55, − 4.58). The mediation analysis showed that mobility (47.9%) explained the largest proportion of the association between multimorbidity and QoL, followed by pain/discomfort (43.5%), sleep/energy (35.0%), negative affect (31.9%), cognition (20.2%), self-care (17.0%), and interpersonal activities (12.0%) (Appendix Table S4).

**Table 1:** Sample characteristics (overall and by multimorbidity)

Characteristic	Overall	Multimorbidity^a^		*P*-value^b^
		No	Yes	
Age				
Mean (SD)	62.4 (16.0)	60.2 (14.4)	65.0 (16.7)	< 0.001
Sex				
Female	52.0	47.6	57.3	< 0.001
Male	48.0	52.4	42.7	
Education				
≤ Primary	57.3	57.7	56.8	0.011
Secondary	35.2	33.8	36.9	
Tertiary	7.5	8.5	6.3	
Wealth				
Poorest	17.2	16.2	18.3	0.005
Poorer	19.0	18.2	19.9	
Middle	19.4	19.1	19.7	
Richer	21.3	21.5	21.0	
Richest	23.2	25.1	21.0	
Marital status				
Currently married/cohabiting	75.5	80.9	69.0	< 0.001
Never married	1.7	1.6	1.8	
Separated/divorced/widowed	22.8	17.4	29.2	
Employment status				
Not employed	42.5	50.5	33.0	< 0.001
Employed	57.5	49.5	67.0	
Social participation^c^				
Physical activity Mean (SD)	21.3 (23.3)	22.7 (23.7)	20.2 (22.8)	< 0.001
High	49.4	53.2	44.8	< 0.001
Moderate	22.8	22.8	22.7	
Low	27.8	24.0	32.4	
Smoking				
Never	58.3	57.0	59.9	< 0.001
Current	35.1	37.7	32.1	
Past	6.6	5.4	8.0	
Affect^d^				
Mean (SD)	21.1 (44.7)	16.0 (41.0)	27.5 (47.3)	< 0.001
Cognition^d^				
Mean (SD)	30.6 (46.1)	25.3 (44.1)	36.9 (46.7)	< 0.001
Interpersonal activity^d^				
Mean (SD)	17.8 (45.5)	14.2 (41.7)	22.7 (49.3)	< 0.001
Mobility^d^				
Mean (SD)	32.6 (46.6)	23.7 (41.8)	43.4 (45.9)	< 0.001
Pain/discomfort^d^				
Mean (SD)	30.3 (44.9)	22.4 (42.0)	39.9 (43.5)	< 0.001
Self-care^d^				
Mean (SD)	10.8 (40.8)	5.7 (30.5)	16.9 (48.6)	< 0.001
Sleep/energy^d^				
Mean (SD)	27.4 (45.2)	19.9 (41.3)	36.6 (45.4)	< 0.001

*SD* Standard deviation

^a^Multimorbidity referred to ≥ 2 chronic physical conditions

^b^*P*-value was based on Chi-squared tests and Student’s *t*-tests for categorical and continuous variables, respectively

^c^Social participation was based on a scale ranging from 0 to 100 with higher scores representing higher levels of social participation

^d^Health status was based on a scale ranging from 0 to 100 with higher scores representing worse health status

**Table 2:** Association between number of chronic physical conditions (or covariates) and quality of life (outcome) estimated by multivariable linear regression

Characteristic		Overall		Age				Sex			
				50–64 years		≥ 65 years		Male		Female	
No. of chronic conditions	0	Ref.		Ref.		Ref.		Ref.		Ref.	
	1	− 2.26***	[− 2.94, − 1.57]	− 2.43***	[− 3.29, − 1.57]	− 1.82*	[− 3.47, − 0.16]	− 2.10***	[− 2.91, − 1.29]	− 2.44***	[− 3.51, − 1.37]
	2	− 5.89***	[− 6.83, − 4.95]	− 6.00***	[− 7.15, − 4.85]	− 5.68***	[− 7.02, − 4.34]	− 6.05***	[− 7.17, − 4.94]	− 5.77***	[− 7.10, − 4.45]
	3	− 8.35***	[− 9.63, − 7.06]	− 8.69***	[− 10.64, − 6.73]	− 7.97***	[− 9.57, − 6.36]	− 8.07***	[− 9.78, − 6.37]	− 8.83***	[− 10.08, − 7.59]
	4	− 10.87***	[− 12.37, − 9.36]	− 11.78***	[− 13.84, − 9.73]	− 9.99***	[− 11.74, − 8.24]	− 11.54***	[− 13.24, − 9.84]	− 10.28***	[− 12.18, − 8.39]
	≥ 5	− 13.48***	[− 15.91, − 11.06]	− 12.60***	[− 16.45, − 8.75]	− 13.42***	[− 15.59, − 11.24]	− 12.82***	[− 15.55, − 10.10]	− 13.63***	[− 16.57, − 10.70]
Age (years)	Per one-year increase	0.04	[− 0.01, 0.08]	0.14**	[0.06, 0.23]	0.01	[− 0.07, 0.09]	0.07*	[0.01, 0.12]	0.04	[− 0.01, 0.09]
Sex	Female	Ref.		Ref.		Ref.					
	Male	0.91*	[0.06, 1.77]	1.21*	[0.26, 2.17]	0.35	[− 0.94, 1.65]				
Education	≤ Primary	Ref.		Ref.		Ref.		Ref.		Ref.	
	Secondary	2.10***	[1.42, 2.78]	1.71***	[0.88, 2.53]	3.22***	[2.10, 4.33]	2.55***	[1.68, 3.42]	1.89***	[0.92, 2.86]
	Tertiary	3.85***	[2.65, 5.06]	3.79***	[2.32, 5.27]	3.99***	[2.09, 5.89]	3.85***	[2.47, 5.23]	4.44***	[2.58, 6.31]
Wealth	Poorest	Ref.		Ref.		Ref.		Ref.		Ref.	
	Poorer	3.86***	[2.76, 4.96]	3.77***	[2.41, 5.14]	3.99***	[2.23, 5.75]	4.04***	[2.70, 5.38]	3.60***	[2.27, 4.94]
	Middle	5.89***	[4.73, 7.04]	6.04***	[4.52, 7.57]	5.74***	[4.15, 7.33]	5.80***	[4.25, 7.36]	5.84***	[4.53, 7.14]
	Richer	7.10***	[6.04, 8.16]	7.05***	[5.55, 8.54]	7.54***	[6.13, 8.95]	7.75***	[6.48, 9.02]	6.36***	[5.03, 7.69]
	Richest	11.34***	[10.21, 12.47]	11.38***	[9.71, 13.05]	11.80***	[9.99, 13.60]	11.57***	[10.16, 12.99]	10.71***	[9.16, 12.26]
Marital status^a^	Currently married	Ref.		Ref.		Ref.		Ref.		Ref.	
	Never married	− 2.48*	[− 4.68, − 0.28]	− 3.88***	[− 6.03, − 1.73]	1.28	[− 3.83, 6.40]	− 3.37*	[− 6.10, − 0.64]	− 1.82	[− 5.00, 1.36]
	Other	− 1.39**	[− 2.38, − 0.40]	− 1.21	[− 2.79, 0.37]	− 1.17*	[− 2.31, − 0.03]	0.80	[− 0.72, 2.33]	− 2.42***	[− 3.60, − 1.23]
Employment	Employed	Ref.		Ref.		Ref.		Ref.		Ref.	
	Unemployed	− 2.05***	[− 2.78, − 1.32]	− 2.52***	[− 3.35, − 1.69]	− 1.98***	[− 3.10, − 0.87]	− 3.33***	[− 4.23, − 2.44]	− 0.99*	[− 1.98, − 0.01]
Social participation^b^	Per one-unit increase	0.15***	[0.12, 0.18]	0.09***	[0.05, 0.13]	0.24***	[0.19, 0.28]	0.15***	[0.11, 0.19]	0.16***	[0.12, 0.21]
Physical activity	High	Ref.		Ref.		Ref.		Ref.		Ref.	
	Moderate	0.05	[− 0.70, 0.80]	− 0.50	[− 1.33, 0.34]	0.75	[− 0.59, 2.08]	− 0.16	[− 1.01, 0.69]	0.35	[− 0.68, 1.38]
	Low	− 2.62***	[− 3.57, − 1.67]	− 1.68**	[− 2.89, − 0.47]	− 3.06***	[− 4.35, − 1.76]	− 2.60***	[− 3.74, − 1.46]	− 2.36***	[− 3.50, − 1.22]
Smoking	Never	Ref.		Ref.		Ref.		Ref.		Ref.	
	Current	− 0.95**	[− 1.64, − 0.25]	− 0.89*	[− 1.77, − 0.01]	− 0.79	[− 1.92, 0.35]	− 0.85*	[− 1.67, − 0.03]	− 1.42*	[− 2.76, − 0.07]
	Past	− 2.91***	[− 3.86, − 1.96]	− 3.59***	[− 4.98, − 2.20]	− 2.07**	[− 3.63, − 0.50]	− 2.93***	[− 4.00, − 1.85]	− 3.38**	[− 5.46, − 1.30]

Models are adjusted for all variables in the respective column and country

*Ref.* Reference category

*p < 0.05, **p < 0.01, ***p < 0.001

^a^Currently married included cohabiting, and the “other” category included separated/divorced/widowed

^b^Social participation was based on a scale ranging from 0 to 100 with higher scores representing higher levels of social participation

Quality of life was based on a scale ranging from 0 to 100 with higher scores representing better quality of life

## Discussion

### Main findings

In this study including 34,129 participants aged ≥ 50 years from six LMICs, a dose–response relationship was found between increasing number of chronic conditions and lower QoL. The association was similar across age groups and sex. In terms of individual chronic conditions, visual difficulty and stroke were associated with particularly low QoL. Country-wise analysis showed that multimorbidity was associated with lower QoL in all six countries included in the study although the estimate for Mexico was not statistically significant. Of note, there was a moderate level of between-country heterogeneity with strongest associations being observed in India and China. Finally, mediation analysis showed that mobility, pain/discomfort, sleep/energy, and affect individually explained more than 30% of the association between multimorbidity and QoL. The finding that QoL decreases with increase in the number of chronic conditions concurs with previous studies mainly from high-income countries [12]. Our study adds to the existing literature by showing for the first time that this association exists in a large multi-country sample of community-dwelling older adults, and by quantifying the extent to which several factors that can be the consequence of chronic conditions or multimorbidity and the cause of low QoL may explain the association between multimorbidity and QoL.

### Interpretation of the findings

The mechanisms as to how multimorbidity contributes to the reduction in QoL is likely due to increasing number of chronic conditions leading to higher physical and mental health impairment, and higher healthcare utilization and expenditure (especially in LMICs), resulting in lower overall QoL [37]. In particular, the accumulating effect of disturbing symptoms of the individual chronic conditions in multimorbidity may lead to greater reduction in QoL. For example, in our study, visual difficulty and stroke were associated with particularly low QoL. This may be due to visual impairment or stroke affecting one’s ability to work or care for themselves (or others), while they may also affect numerous casual activities such as reading, socializing, and pursuing hobbies [38].

In our study, we were able to quantify the individual contribution of potential mediators in the association between multimorbidity and QoL, and this is particularly important as it provides detailed information on what mechanisms may underlie this association. Specifically, we found that mobility explains nearly 50% of the association, followed by pain/discomfort, sleep/energy, and affect which all explained more than 30% of the association. Cognition, self-care, and interpersonal activities also explained 12.0–20.2% of the association. Factors such as mobility limitation, pain/discomfort, and sleep problems are frequent in people with chronic conditions due to the symptoms per se (e.g., mobility limitations in stroke, pain in arthritis, sleep problems in chronic lung disease or asthma due to breathing problems) [33, 39]. Furthermore, longitudinal studies have found that multimorbidity precedes psychological conditions such as anxiety [40] and depression [41], and this may be explained by factors such as chronic pain, frailty, symptom burden, and functional impairment [41]. All these conditions (especially mobility limitations and pain) may also directly lead to loss in functional independence [42], which in our study may be reflected in difficulty in self-care. Previous studies have also shown that multimorbidity is associated with lower social participation and impaired cognition [22, 33] and this is likely owing to the above mentioned factors (e.g., chronic pain, symptom burden etc.). In turn, all the mediators assessed in our study have been reported to reduce QoL [43–45].

Finally, we found a moderate level of between-country heterogeneity in the association between multimorbidity and QoL. Although the reason for this can only be speculated, it is possible for factors such as difference in the chronic conditions that constitute multimorbidity [16] or quality of health care between countries to have contributed to this between-country heterogeneity. For example, a previous SAGE study showed that the prevalence of hypertension (which was not strongly associated with QoL in our study) is particularly high in countries where the magnitude of the association was less pronounced (e.g., Mexico, South Africa). Furthermore, given that mobility and pain were the main factors that explain the multimorbidity/QoL relationship, availability of rehabilitation services, wheelchairs, or pain killers, for example, are likely to vary substantially between countries and this can lead to heterogeneity in the magnitude of the association between multimorbidity and QoL. However, clearly, more research including more countries is necessary to understand the underlying factors of the heterogeneity observed.

### Public health and clinical implications

Considering these findings, public health and medical practitioners should aim to address mobility limitations (regarding both physical mobility and access to mobility aids), pain, sleep problems, mental health, functional limitations, and social support through targeted interventions and public health policy to improve QoL among older adults in LMICs with multimorbidity. Such interventions may wish to include mind–body exercises (e.g., tai-chi, yoga) which have been shown to improve mental health complications, mobility and QoL per se [46]. Moreover, mind–body exercise has been found to be suitable for those with chronic conditions [46, 47]. It is also worth noting here that the individual condition “visual difficulty” was particularly strongly associated with lower levels of QoL in the present study. It may also be prudent to target those with visual difficulties with similar interventions to those for multimorbidity to improve QoL [48]. Apart from this, decreasing the economic burden of healthcare in LMICs to reduce decreases in QoL due to financial burden is highly warranted [49].

### Strengths and limitations

The strengths of the study include the use of large nationally representative datasets and the use of the WHOQoL instrument to measure QoL, which has been shown to have good internal consistency and acceptable convergent validity [18, 19]. However, the results of this study should be considered within its limitations. First, this is a cross-sectional analysis, which does not allow us to establish a causal direction; it is possible that relations are bi-directional. Second, multimorbidity was measured using a unit increase in the number of chronic conditions, but the number of chronic conditions of an individual may not necessarily reflect the severity of disease burden. Relatedly, although our list of chronic conditions included a variety of conditions which are common in old age, it is possible for the results to have differed with the use of a different list of chronic conditions. Finally, mediation and confounding are identical statistically and can be distinguished only on conceptual grounds [50]. While many of the potential mediators assessed in this study can be conceptualized as mediators, it is possible for the mediating effect to be an overestimation given the various ways in which multimorbidity, QoL, and the mediators can be intertwined. In addition, there were some conceptual overlaps with some of the mediators and the items of the 8-item WHOQoL instrument, and this could have also accentuated the mediated percentage.

## Conclusion and implications

This study showed a significant inverse dose–response relationship between increasing number of chronic physical conditions and QoL among older adults in LMICs. Furthermore, some potentially important mediators such as mobility limitations, pain, and mental health problems were identified. Future intervention studies (ideally randomized controlled studies) with long follow-up periods are warranted to examine whether addressing the identified mediators can improve QoL in older people with multimorbidity in LMICs.

## Supplementary Information

Below is the link to the electronic supplementary material.

Supplementary file1 (DOCX 27 KB)

## Data Availability

The data that support the findings of this study are available on request from the corresponding author.
