# Supplementary material for: Chronic physical conditions, physical multimorbidity, and quality of life among adults aged ≥ 50 years from six low- and middle-income countries
Source: Qual Life Res. 2022 Dec 26;32(4):1031–41. doi: 10.1007/s11136-022-03317-6 (PMC10063492; doi:10.1007/s11136-022-03317-6)
Supplement: Supplementary file 1 — Supplementary file1 (DOCX 27 KB) [file 11136_2022_3317_MOESM1_ESM.docx]

**APPENDIX**

| **Table S1** Details on the diagnosis of chronic conditions | | |
| --- | --- | --- |
| Condition | (a) Self-reported diagnosis | (b) Symptom-based algorithm or other method of diagnosis^a^ |
| Angina | Have you ever been diagnosed with angina or angina pectoris (a heart disease)? | Rose questionnaire [1] |
| Arthritis | Have you ever been diagnosed with/told you have arthritis (a disease of the joints, or by other names rheumatism or osteoarthritis)? | Affirmative answers to all four of the following: 1. During the last 12 months, have you experienced pain, aching, stiffness or swelling in or around the joints (e.g., in arms, hands, legs or feet) which were not related to an injury and lasted for more than a month? 2. During the last 12 months, have you experienced stiffness in the joint in the morning after getting up from bed, or after a long rest of the joint without movement? 3. Did this stiffness last for less than 30 minutes? 4. Did this stiffness go away after exercise or movement in the joint? |
| Asthma | Have you ever been diagnosed with asthma (an allergic respiratory disease)? | 1. During the last 12 months, have you experienced attacks of wheezing or whistling breathing? (Yes)  **AND** 2. “Yes” to at least one of the following (past 12 months): (a) Have you experienced an attack of wheezing that came on after you stopped exercising or some other physical activity? (b) Have you had a feeling of tightness in your chest? (c) Have you woken up with a feeling of tightness in your chest in the morning or any other time? (d) Have you had an attack of shortness of breath that came on without an obvious cause when you were not exercising or doing some physical activity? |
| Chronic lung disease | Have you ever been diagnosed with chronic lung disease (emphysema, bronchitis, COPD)? | 1. During the last 12 months, have you experienced any shortness of breath at rest (while awake)?  (Yes) **OR** 2. “Yes” to both of the following (past 12 months): (a) Have you experienced any coughing or wheezing for 10 minutes or more at a time? (b) Have you experienced any coughing up of sputum or phlegm on most days of the month for at least 3 months? |
| Diabetes | Have you ever been diagnosed with diabetes (high blood sugar)? (not including diabetes associated with a pregnancy) | NA |
| Hypertension | Have you ever been diagnosed with high blood pressure (hypertension)? | Blood pressure was measured three times with a one-minute interval with the use of a wrist blood pressure monitor (Medistar Wrist Blood Pressure Model S) and the mean value of the three measurements was calculated. Hypertension was defined as having at least one of the following: systolic blood pressure ≥140 mmHg; diastolic blood pressure ≥90 mmHg. |
| Stroke | Have you ever been told by a health professional that you have had a stroke? | NA |

For all chronic conditions, we assumed that the individual had the condition if they fulfilled at least one of the following: (a) affirmative answer to self-reported diagnosis or (b) symptom-based algorithm or other method of diagnosis.

^a^ These algorithms have been used in previous publications [2, 3] and those of arthritis, asthma, and chronic lung disease have been validated [2, 4].

[1] Rose GA. The diagnosis of ischaemic heart pain and intermittent claudication in field surveys. Bull World Health Organ. 1962;27: 645-658.

[2] Arokiasamy P, Uttamacharya, Kowal P, et al. Chronic Noncommunicable Diseases in 6 Low- and Middle-Income Countries: Findings From Wave 1 of the World Health Organization's Study on Global Ageing and Adult Health (SAGE). Am J Epidemiol. 2017;185: 414-428.

[3] Garin N, Koyanagi A, Chatterji S, et al. Global Multimorbidity Patterns: A Cross-Sectional, Population-Based, Multi-Country Study. J Gerontol A Biol Sci Med Sci. 2016;71: 205-214.

[4] Moussavi S, Chatterji S, Verdes E, Tandon A, Patel V, Ustun B. Depression, chronic diseases, and decrements in health: results from the World Health Surveys. Lancet. 2007;370: 851-858.

| **Table S2** Questions used to assess health status | |
| --- | --- |
| **Mobility** | (1) Overall in the last 30 days, how much difficulty did you have with moving around? |
|  | (2) Overall in the last 30 days, how much difficulty did you have in vigorous activities (`vigorous activities’ require hard physical effort and cause large increases in breathing or heart rate)? |
| **Self-care** | (1) Overall in the last 30 days, how much difficulty did you have with self- care, such as bathing/washing or dressing yourself? |
|  | (2) Overall in the last 30 days, how much difficulty did you have in taking care of and maintaining your general appearance (e.g. grooming, looking neat and tidy)? |
| **Pain and discomfort** | (1) Overall in the last 30 days, how much of bodily aches or pains did you have?  (2) Overall in the last 30 days, how much bodily discomfort did you have? |
| **Cognition** | (1) Overall in the last 30 days, how much difficulty did you have with concentrating or remembering things? |
|  | (2) Overall in the last 30 days, how much difficulty did you have in learning a new task (for example, learning how to get to a new place, learning a new game, learning a new recipe etc.)? |
| **Interpersonal activities** | (1) Overall in the last 30 days, how much difficulty did you have with personal relationship or participation in the community? |
|  | (2) Overall in the last 30 days, how much difficulty did you have in dealing with conflicts and tensions with others? |
| **Sleep and energy** | (1) Overall in the last 30 days, how much of a problem did you have with sleeping, such as falling asleep, waking up frequently during the night or waking up too early in the morning? |
|  | (2) Overall in the last 30 days, how much of a problem did you have due to not feeling rested and refreshed during the day (e.g. feeling tired, not having energy)? |
| **Affect** | (1) Overall in the last 30 days, how much of a problem did you have with feeling sad, low or depressed? |
|  | (2) Overall in the last 30 days, how much of a problem did you have with worry or anxiety? |

| **Table S3** Questions used to assess social participation |
| --- |
| How often in the last 12 months have you ... |
| (1) attended any public meeting in which there was discussion of local or school affairs? |
| (2) met personally with someone you consider to be a community leader? |
| (3) attended any group, club, society, union or organizational meeting? |
| (4) worked with other people in your neighborhood to fix or improve something? |
| (5) had friends over to your home? |
| (6) been in the home of someone who lives in a different neighbourhood than you do or had them in your home? |
| (7) socialized with coworkers outside of work? |
| (8) attended religious services (not including weddings and funerals)? |
| (9) gotten out of the house/your dwelling to attend social meetings, activities, programs or events or to visit friends or relatives? |

The answer option to these questions were ‘never (coded=1)’, ‘once or twice per year (coded=2)’, ‘once or twice per month (coded=3)’, ‘once or twice per week (coded=4)’, and ‘daily (coded=5)’.

| **Table S4** Mediators in the association between multimorbidity and quality of life (outcome) | | | | | | | |
| --- | --- | --- | --- | --- | --- | --- | --- |
| Mediator | Total effect | P-value | Direct effect | P-value | Indirect effect | P-value | %Mediated |
| Affect | -6.31 [-7.03,-5.58] | <0.001 | -4.29 [-5.04,-3.54] | <0.001 | -2.01 [-2.32,-1.71] | <0.001 | 31.9 |
| Cognition | -6.34 [-7.16,-5.52] | <0.001 | -5.06 [-5.88,-4.24] | <0.001 | -1.28 [-1.51,-1.06] | <0.001 | 20.2 |
| Interpersonal activities | -6.34 [-7.13,-5.54] | <0.001 | -5.58 [-6.38,-4.77] | <0.001 | -0.76 [-0.96,-0.56] | <0.001 | 12.0 |
| Mobility | -6.33 [-7.14,-5.52] | <0.001 | -3.29 [-4.10,-2.49] | <0.001 | -3.03 [-3.35,-2.71] | <0.001 | 47.9 |
| Pain/discomfort | -6.33 [-7.12,-5.54] | <0.001 | -3.58 [-4.37,-2.79] | <0.001 | -2.75 [-3.07,-2.44] | <0.001 | 43.5 |
| Self-care | -6.34 [-7.18,-5.50] | <0.001 | -5.26 [-6.09,-4.43] | <0.001 | -1.08 [-1.27,-0.89] | <0.001 | 17.0 |
| Sleep/energy | -6.33 [-7.10,-5.56] | <0.001 | -4.12 [-4.91,-3.33] | <0.001 | -2.21 [-2.48,-1.95] | <0.001 | 35.0 |

Estimates are b-coefficients [95% confidence intervals].

Quality of life was based on a scale ranging from 0 to 100 with higher scores representing better quality of life.

Models are adjusted for age, sex, education, wealth, marital status, unemployment, social participation, physical activity, smoking, and country.
